# Supplementary material for: Needle guidance with Doppler-tracked polarization-sensitive optical coherence tomography
Source: J Biomed Opt. 2023 Oct 4;28(10):102910. doi: 10.1117/1.JBO.28.10.102910 (PMC10548115; doi:10.1117/1.JBO.28.10.102910)
Supplement: Supplementary file 1 [file JBO_028_102910_SD001.pdf]

# Needle guidance with Doppler-tracked polarization-sensitive optical coherence tomography

## [supplementary information]

Danielle J. Harper[a,b,*], Yongjoo Kim[a,b], Alejandra Gómez-Ramírez[a,c] and Benjamin J. Vakoc[a,b,d]

[a]Wellman Center for Photomedicine, Massachusetts General Hospital, 40 Blossom Street, Boston, MA 02114, USA
[b]Harvard Medical School, 25 Shattuck Street, Boston, MA 02115, USA
[c]School of Physics, Universidad Nacional de Colombia sede Medellín, 3840-Medellín-050034, Colombia
[d]Harvard-MIT Division of Health Sciences and Technology, 77 Massachusetts Avenue, Cambridge, MA 02139, USA

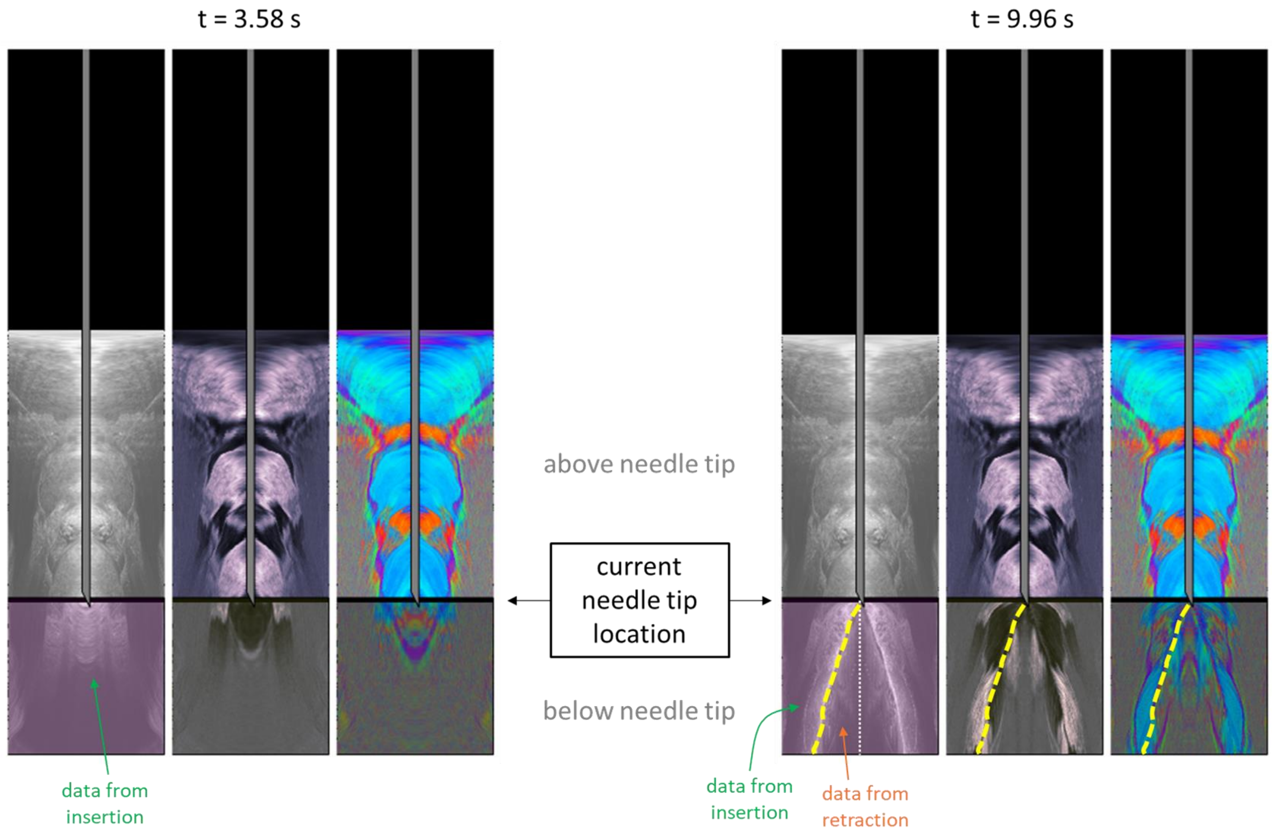


**Figure S1:** Two still frames from the movie shown in Supplementary Video 1 with annotations. At t = 3.58 s (during insertion), all data below the needle tip has a high signal-to-noise ratio as the forward-looking needle has not yet made a hole in the tissue. At t = 9.96 s (during retraction), the needle tip has returned to the same location, but the forward-looking view now sees a much noisier signal due to the hole that the needle has made, as shown in the needle-referenced maps in Fig. 2 (b-d). However, since the tissue type at this location was known during insertion, when we update the visualization with the real-time view, we do not delete this prior knowledge. Instead, we show the data from both timepoints; the separation indicated with the yellow dashed line.
